# Induction of endogenous Type I interferon within the central nervous system plays a protective role in experimental autoimmune encephalomyelitis

**DOI:** 10.1007/s00401-015-1418-z

**Published:** 2015-04-14

**Authors:** Reza Khorooshi, Marlene Thorsen Mørch, Thomas Hellesøe Holm, Carsten Tue Berg, Ruthe Truong Dieu, Dina Dræby, Shohreh Issazadeh-Navikas, Siegfried Weiss, Stefan Lienenklaus, Trevor Owens

**Affiliations:** Department of Neurobiology Research, Institute of Molecular Medicine, University of Southern Denmark, J.B. Winsloewsvej 25, 5000 Odense C, Denmark; Department of Biomedicine, Aarhus University, Aarhus, Denmark; Neuroinflammation Unit, BRIC, University of Copenhagen, Copenhagen, Denmark; Department of Molecular Immunology, Helmholtz Centre for Infection Research, Braunschweig, Germany

**Keywords:** Interferon-beta, Interferon-alpha, Microglia, Macrophages, Poly I:C, EAE

## Abstract

The Type I interferons (IFN), beta (IFN-β) and the alpha family (IFN-α), act through a common receptor and have anti-inflammatory effects. IFN-β is used to treat multiple sclerosis (MS) and is effective against experimental autoimmune encephalomyelitis (EAE), an animal model for MS. Mice with EAE show elevated levels of Type I IFNs in the central nervous system (CNS), suggesting a role for endogenous Type I IFN during inflammation. However, the therapeutic benefit of Type I IFN produced in the CNS remains to be established. The aim of this study was to examine whether experimentally induced CNS-endogenous Type I IFN influences EAE. Using IFN-β reporter mice, we showed that direct administration of polyinosinic–polycytidylic acid (poly I:C), a potent inducer of IFN-β, into the cerebrospinal fluid induced increased leukocyte numbers and transient upregulation of IFN-β in CD45/CD11b-positive cells located in the meninges and choroid plexus, as well as enhanced IFN-β expression by parenchymal microglial cells. Intrathecal injection of poly I:C to mice showing first symptoms of EAE substantially increased the normal disease-associated expression of IFN-α, IFN-β, interferon regulatory factor-7 and IL-10 in CNS, and disease worsening was prevented for as long as IFN-α/β was expressed. In contrast, there was no therapeutic effect on EAE in poly I:C-treated IFN receptor-deficient mice. IFN-dependent microglial and astrocyte response included production of the chemokine CXCL10. These results show that Type I IFN induced within the CNS can play a protective role in EAE and highlight the role of endogenous type I IFN in mediating neuroprotection.

**Electronic supplementary material:**

The online version of this article (doi:10.1007/s00401-015-1418-z) contains supplementary material, which is available to authorized users.

## Introduction

Interferon (IFN)-β and IFN-α constitute the Type I IFN family, members of which play a central role in antiviral immune responses and in regulation of inflammation [3, 30]. They signal through a common receptor (IFNAR) to activate transcription of several genes including interferon regulatory factor 7 (IRF7) and IRF9, which are also involved in the induction of Type I IFN [14, 30]. Importantly, IFN-β is used as a first-line treatment for multiple sclerosis (MS).

Type I IFN are induced by engagement of innate immune receptors, including toll-like receptors (TLR) and retinoic acid-inducible gene (RIG) I-like helicases (RLH). Innate receptors induce responses by detecting molecular structures shared by many pathogens as well as endogenous agonists associated with tissue damage [19]. Pathogen-derived and experimental agonists such as the synthetic double-stranded RNA analog polyinosinic–polycytidylic acid (poly I:C) engage TLR3, melanoma differentiation-associated protein 5 (MDA5) and RIG-I and lead to cytokine synthesis and secretion, including IFN-α and IFN-β (IFN-α/β) [38].

Peripheral administration of poly I:C has been shown to suppress the progression of experimental autoimmune encephalomyelitis (EAE) [7, 36]. Whether the suppressive effect of poly I:C on EAE involved CNS or peripheral action of IFN-α/β is not clear [7, 36]. Peripheral IFN-α/β may access the inflamed CNS [30]. IFN-β is dramatically increased in the CNS of mice with EAE [31], and Type I IFN response has been implicated in regulation of EAE [31, 32, 35]. Together, these findings suggest an important role for endogenous type I IFN within the CNS. However, there is a paucity of information about cellular sources and the action of IFN-α/β produced in the CNS.

The aim of this study was to examine the therapeutic role of IFN-α/β produced in the CNS during EAE. Direct administration of poly I:C into the cerebrospinal fluid (CSF) via the cisterna magna transiently induced IFN-β expression by myeloid cells in meninges and choroid plexus, and increased the expression of IFN-α/β by microglia. Therapeutic inhibition of established EAE correlated temporally with IFN-α/β expression, and was IFNAR1 and IFN-α/β dependent. Astrocytes and microglia upregulated IFN-response genes and the IFNAR1-dependent chemokine CXCL10. IFN-α/β produced within the CNS, therefore, mediates endogenous neuroprotection.

## Materials and methods

### Mice

C57BL/6 mice were purchased from Taconic (Taconic Europe, Ry, Denmark). IFNAR1-KO mice (C57BL/6 background) [28] and transgenic GFAP-EGFP mice (FVB background) [29] originally from Drs. Marco Prinz, University of Freiburg, Germany and Helmut Kettenmann, Max-Delbrück-Center for Molecular Medicine, Berlin, Germany, respectively, were bred and housed in the Biomedical Laboratory, University of Southern Denmark. Experiments were conducted in accordance with the national ethical committee (Animal Experiments Inspectorate under Danish Ministry of Food, Agriculture and Fisheries, The Danish Veterinary and Food Administration) (approval number 2012-15-2934-00110).

IFN-β^mob/mob^ mice [33] were obtained from Jackson Laboratory and Albino (C57BL/6-Tyr^c−2J^) IFN-β^+/Δβ−luc^ mice (IFN-β-luciferase reporter mice [21]) were bred and housed at the Department of Molecular Immunology, Helmholtz Centre for Infection Research, Braunschweig, Germany. Experiments using IFN-β^+/Δβ−luc^ mice were performed under approval number 33.9-42502-04-12/0968 of local authority Niedersächsisches Landesamt für Verbraucherschutz und Lebensmittelsicherheit (LAVES).

### EAE induction

C57BL/6 and IFNAR1-deficient mice were immunized with MOG p35-55, kindly provided by Mogens Nielsen at the Centre for Experimental Bioinformatics, Department of Biochemistry and Molecular Biology, University of Southern Denmark. Emulsions of MOG p35-55 (100 μg) and complete Freund’s adjuvant with heat-inactivated *Mycobacterium tuberculosis* (200 μg; Difco Laboratories, Detroit) were injected subcutaneously. Animals received an intraperitoneal injection of pertussis toxin (0.3 μg; Sigma-Aldrich, Brøndby, Denmark) at the time of immunization and 2 days post-immunization, as described previously [26]. Mice were monitored for loss of body weight and EAE symptoms. The EAE grades were as follows: Grade 0, no signs of disease; Grade 1, weak or hooked tail; Grade 2, floppy tail indicating complete loss of tonus in tail; Grade 3, floppy tail and hind limb paresis, Grade 4: floppy tail and unilateral hind limb paralysis; Grade 5, floppy tail and bilateral hind limb paralysis. For ethical reasons, mice were not allowed to reach grades higher than 5.

### Intrathecal injection

Mice were anesthetized using isoflurane and the back of the head was shaved. A 30-gauge needle (bent at 55°, 2 mm from the tip) attached to a 50-µl Hamilton syringe was inserted between the skull and the cervical vertebra into the intrathecal space of the cisterna magna. Mice received intrathecal injection (10 µl) of poly I:C (Sigma-Aldrich, Copenhagen, Denmark) at 0, 3, 1, 3 and 10 mg/ml, or PBS. Intrathecal injection allows delivery of substances to the CNS with minimal trauma [1, 11, 25]. The mice were euthanized 6, 18 and 72 h post-injection with an overdose of sodium pentobarbital and subsequently perfused transcardially with PBS. For flow cytometric analysis, brains and spinal cords were removed into ice cold Ca^2+^/Mg^2+^ free Hanks balanced salt solution (HBSS) before being dissociated.

For histology, mice were additionally perfused with 4 % PFA in PBS. After removal, brains and spinal cords were post-fixed in 4 % PFA, immersed in 30 % sucrose in PBS at 4 °C overnight, frozen with liquid nitrogen and stored at −80 °C until sections were cut on a cryostat.

### Flow cytometric cell sorting

Brains and spinal cords from transgenic GFAP-EGFP mice were dissociated using the papain-based neural tissue dissociation kit (Miltenyi Biotec, Germany). Myelin was separated from the cells on a discontinuous Percoll gradient (GE Healthcare Biosciences AB, Uppsala, Sweden) and the cells were washed and incubated with blocking solution containing HBSS, FBS (Sigma-Aldrich), anti-Fc receptor antibody (BD Biosciences, Brøndby, Denmark), hamster IgG (Jackson ImmunoResearch, West Grove, PA, USA), and sodium azide. The cells were then labeled with phycoerythrin (PE)-conjugated anti-CD45 (BD Biosciences) for 15 min at 4 °C and propidium iodide (PI, Sigma-Aldrich) to detect microglia/macrophages and non-viable cells, respectively.

Cells were sorted using a FACSVantage SE DiVa cell sorter (BD Biosciences). Astrocytes were defined as EGFP positive and CD45 negative (Fig. 6b). Microglia were defined as EGFP negative and CD45^dim^. Sorted astrocytes and microglia were re-analyzed by flow cytometry and quantitative real-time RT-PCR to verify purity.

### Quantitative real-time PCR (qRT-PCR) of sorted cells

RNA extraction was performed using an ABI PRISM™ 6700 Automated Nucleic Acid Workstation (Applied Biosystems, Foster City, CA, USA) according to the manufacturer’s protocol for total RNA purification from cultured cells with including (optional) DNAse treatment or using a Trizol protocol as described previously [32]. The RNA was converted into cDNA using high-capacity cDNA reverse transcription kits (Applied Biosystems). qRT-PCR was performed using an ABI Prism 7300 sequence detection system (Applied Biosystems). 18S rRNA was used for normalization of gene expression [18]. *C*_t_ values were determined and Δ*C*_t_ values were calculated by subtracting the average of *C*_t_ values of gene of interest from *C*_t_ value for the 18S. The relative gene expression was then calculated using ${2^{ - \Delta {C_{\text{t}}}}}$ method.

The following primer and probe sequences were used: CD68 (Forward GCTCCCTGTG TGTCTGATCTTG, Reverse GCCTTTTTGTGAGGACAGTCTTC, Probe CCGCTTATAGCCCAAGGA MGB), GFAP (Forward ACAGACTTTCTCCAACCTCCAGAT, Reverse GCCTTCTGACACGGATTTGGT, Probe CGAGAAACCAGCCTGG MGB), IRF-7 (Forward CACCCCCATCT TCGACTTCA, Reverse CCAAAACCCAGGTAGATGGTGTA, Probe CACTTTCTTCCGAG AACT MGB), IFN-β (Forward GCGTTCCTGCTGTGCTTCTC, Reverse TTGAAGTCCGCCCTGTAGGT, Probe CGGAAATGTCAGGAGCT MGB), IFN-α(B+6+12+14) (Forward AGGATGTGACCTGCCTCAGACT, Reverse GCTGGGCATCCACCTTCTC, Probe CTCTCTCCTGCCTGAAG MGB), CCL2 (Forward TCTGGGCCTGCTGTTCACA, Reverse ACTCATT GGGA TCATCTTGCT, Probe CTCAGCCAG ATGCAGTT MGB), CXCL10 (Forward GCCGT CATTTTCTGCCTCAT, Reverse GGCCCGTCATCGATATGG, Probe GGACTCAAGGGATCC MGB), IRF-9 (Forward ACAACTGAGGCCACCATTAGAGA, Reverse CACCACTCGGCCACCATAG, Probe TGAACTCAGACTACTCGCT MGB), IL-10 (Forward GGTTGCCAAGCCTTATCGGA, Reverse ACCTGCTCCACTGCCTTGCT, Probe TGAGGCGCTGTCATCGATTTCTCCC TAMRA) IL-17A (Forward CTCCAGAAGGCCCTCAGACTAC, Reverse TGTGGTGGTCCAGCTTTCC, Probe ACTCTCCACCGCAATGA MGB), IFN-γ (Forward CATTGAAAGCCTAGAAAGTCTGAATAAC, Reverse TGGCTCTGCAGGATTTTCATG, Probe TCACCATCCTTTTGCCAGTTCCTCCAG MGB).

qRT-PCR analysis of mRNA for the astrocyte marker GFAP and the myeloid marker CD68 was used to verify purities. Sorted astrocytes with relative CD68/18S levels above 10 were omitted and vice versa for microglia and GFAP.

### Immunostaining

For identifying cellular localization of IFN-β, we used IFN-β/YFP reporter mice. Tissue sections (16 µm) were rinsed in PBS containing 0.2 % Triton X-100 (PBST). The sections were incubated in blocking solution containing PBST and 3 % BSA, and stained either with rabbit anti-GFP antibody (ab6556; Abcam), PE-conjugated rat anti-mouse CD45 (BD Biosciences), Cy3-conjugated anti-GFAP or anti-Mac-1/CD11b (MCA711, Serotec, Oxford, UK). After 3 washes in PBST, sections were incubated with biotinylated goat anti-rabbit (Abcam), Alexa-569 goat anti-rat or streptavidin-HRP. GFP signal was amplified with TSA fluorescein kits (PerkinElmer) according to the manufacturer’s instructions. Sections were incubated with DAPI and mounted using gelvatol [17].

Images were acquired using an Olympus DP71 digital camera mounted on an Olympus BX51 microscope (Olympus, Ballerup, Denmark) and with Olympus FV1000MPE Confocal and Multiphoton Laser Scanning Microscope, Danish Molecular Biomedical Imaging Center (DaMBIC), University of Southern Denmark. Images were combined using Adobe Photoshop CS3 (Adobe Systems Denmark A/S, Copenhagen, Denmark) to visualize double-labeled cells.

### Detection of luciferase

For in vivo imaging, IFN-β^+/Δβ−luc^ mice were injected (i.v.) with D-luciferin (150 mg/kg), anesthetized using isoflurane and monitored using an IVIS 200 imaging system (CaliperLS). Photon flux was quantified using Living Image 4.4 software (CaliperLS).

## Results

### Intrathecal poly I:C transiently induced IFN-β in the CNS

**Fig. 1:**
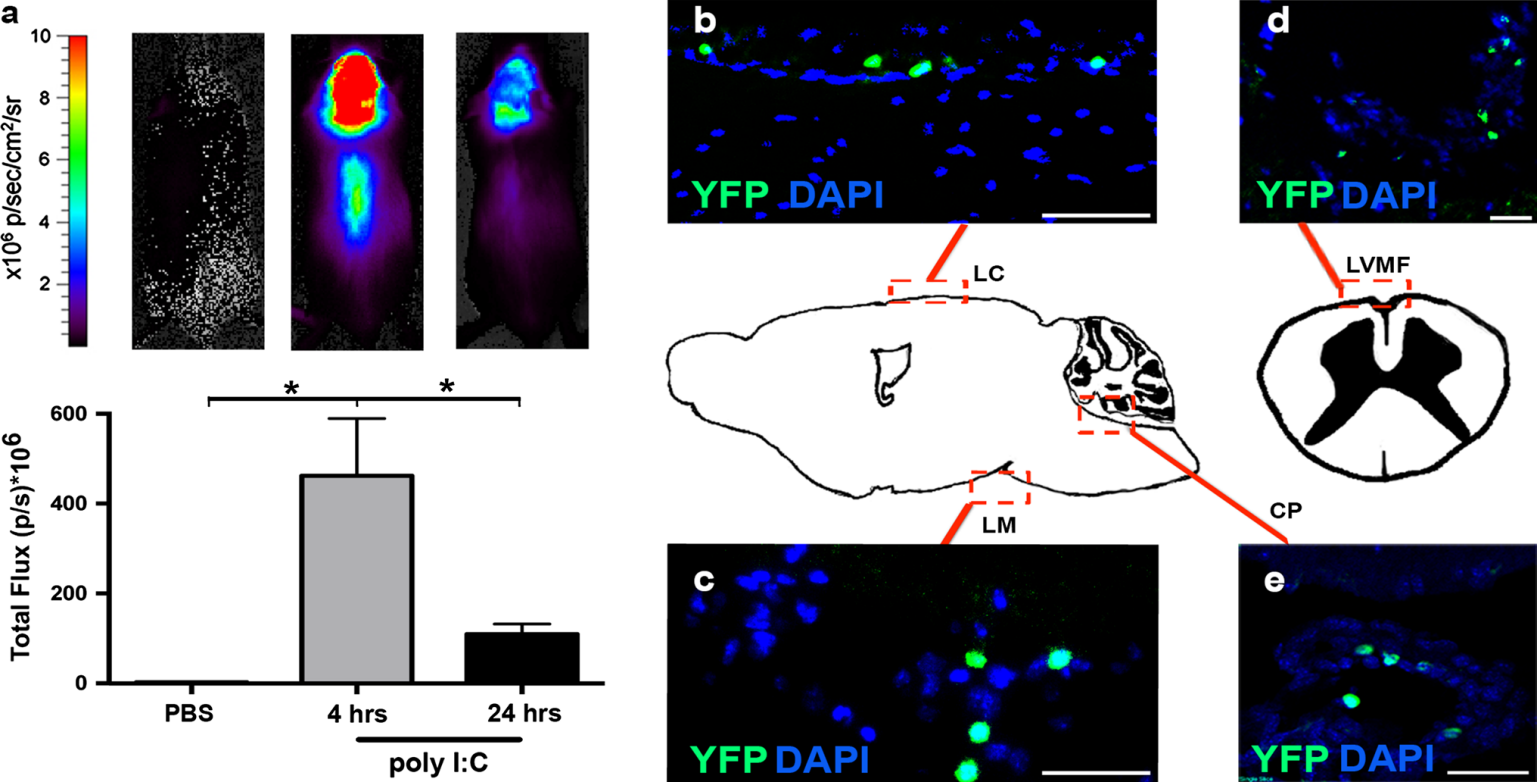
Intrathecal poly I:C induced IFN-β in the CNS. **a** IFN-β reporter mice received poly I:C by intrathecal injection and luciferase activity was visualized after 4 and 24 h. Poly I:C induced IFN-β in the brain and spinal cord at 4 and 24 h. IFN-β response was much stronger at 4 h than at 24 h. *Bar graphs* depict the quantification of luciferase activity at indicated time points (*n* = 4). Data were analyzed by two-tailed nonparametric Student’s *t* test followed by Mann–Whitney test. Results are presented as mean ± SEM. *P < 0.05. Distribution of IFN-β-producing cells in the CNS. IFN-β/YFP+ staining (*green*) was observed in meninges around brain (**b**, **c**) and spinal cord (**d**) as well as choroid plexus (**e**), as indicated by *red boxes* on the central schematic. Nuclei were stained with DAPI (*blue*). *Scale bars* 50 µm (**b**–**e**). *CP* choroid plexus in fourth ventricle, *LC* leptomeningeal cortex, *LM* leptomeningeal midbrain, *LVMF* leptomeningeal ventral median fissure. Original magnification 10×

Poly I:C was injected into CSF via the cisterna magna of reporter mice that express a luciferase gene under the control of the IFN-β promoter [21]. In vivo imaging revealed CNS-restricted expression of IFN-β in response to poly I:C (Fig. 1a). The luciferase signal was expressed in brain and spinal cord and extended rostrally and caudally from the site of injection. Expression was strongest at 4 h, was significantly reduced at 24 h (Fig. 1), and was undetectable at 48 h (not shown). The induction of IFN-β expression was dose dependent, and optimally induced by 10 μg poly I:C (Supplementary Fig. 1).

### IFN-β was expressed by cells in the meninges and choroid plexus

In preference to luciferase staining [16], we used IFNβ/yellow fluorescent protein (YFP) knock-in (IFN-β^mob/mob^) mice, which allow easy detection of IFN-β expression directly at the cellular level [33]. Six hours after poly I:C injection into the cisterna magna, brains and spinal cords were examined for IFN-β expression using an anti-GFP antibody which detects YFP [33]. Sparse IFN-β/YFP-expressing cells were distributed in meninges in the brain (Fig. 1b, c), in spinal cord (Fig. 1d), and in choroid plexus (Fig. 1e). Despite rigorous searching, we could not find YFP+ cells within CNS parenchyma. IFN-β/YFP staining was not detected in CNS from PBS-injected mice. Control sections treated without primary antibody or with isotype-matched primary antibodies showed no staining (not shown). Preliminary analysis of liver and thymus showed no discernible change in the constitutive IFN-β signal [21], supporting a CNS-restricted effect of poly I:C (Supplementary Fig. 2).

### IFN-β was expressed by CD45+ and Mac1/CD11b^+^ cells

**Fig. 2:**
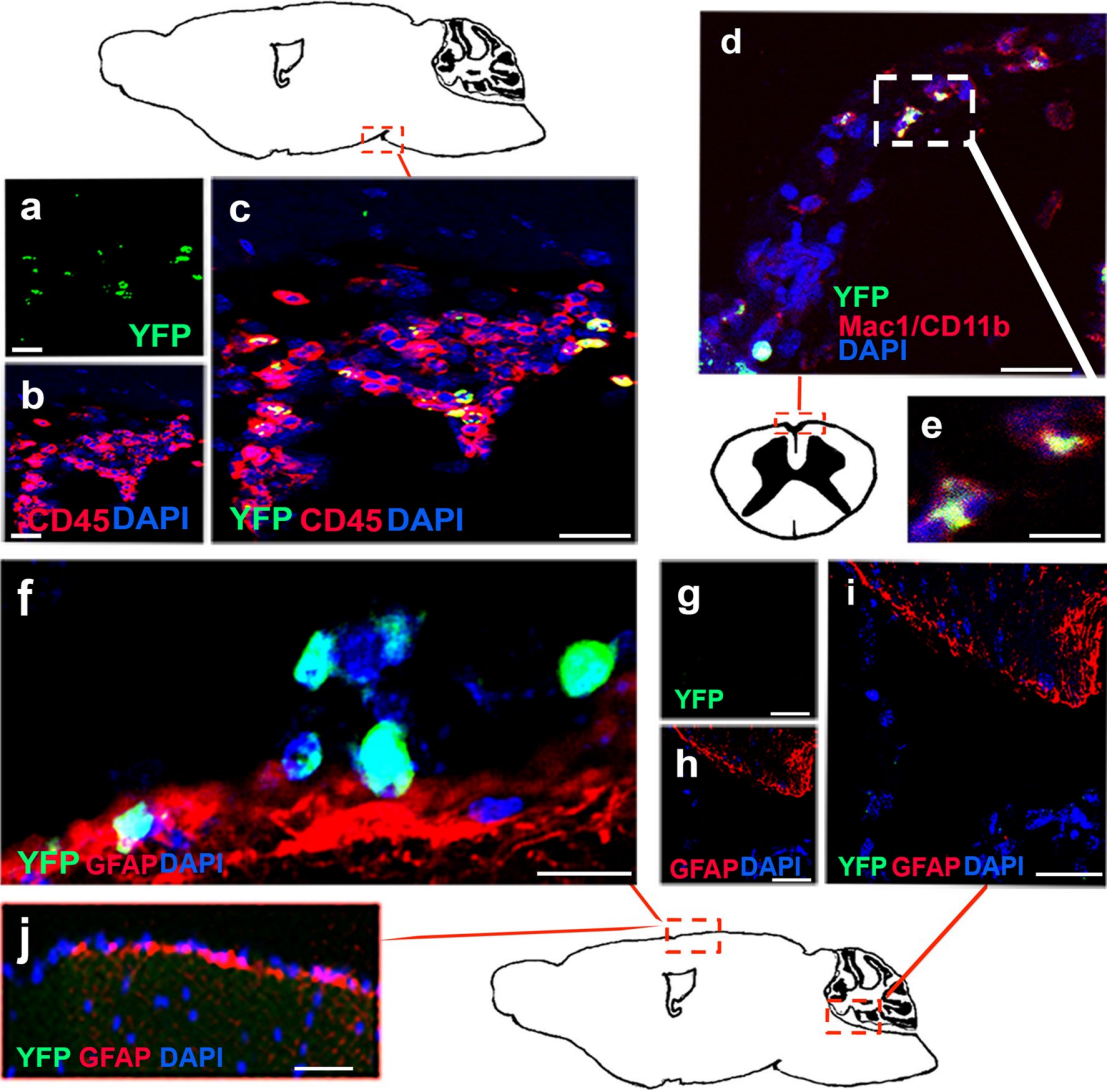
Cellular sources of IFN-β in the CNS. **a**–**c** Double immunostaining showed that IFN-β/YFP+ was induced in cells (*green*) that co-localized both with CD45 (*red*) and **d** with Mac1/CD11b (*red*), in mice treated with poly I:C. **e** Higher magnification of double positive YFP+ Mac1/CD11b+ cells in **d** (*white box*, magnification 20×). **f** Poly I:C-induced IFN-β/YFP+ (*green*) stained cells within meninges did not co-localize with GFAP+ staining (*red*). DAPI (*blue*) shows nuclear staining. **g**–**i** IFN-β/YFP+ staining was not induced in CNS from a PBS-treated mouse. **j** IFN-β/YFP+ staining was absent in sections incubated with normal rabbit antibody. *Scale bars* 50 µm (**a**–**d**, **g**–**j**) and 20 µm (**e**, **f**). Original magnification was 10×

To identify cellular sources of IFN-β, we double-stained IFN-β/YFP+ cells for GFAP and Mac1/CD11b. Double immunostaining showed that IFN-β/YFP+ cells at 6 h post-poly I:C injection were positive for CD45 and Mac1/CD11b (Fig. 2a–e). IFN-β/YFP+ cells did not co-localize with GFAP (Fig. 2f). This identifies extraparenchymal myeloid cells as a source of poly I:C-induced IFN-β.

### Parenchymal microglia and CD45^high^ leukocytes are sources of IFN-α/β in CNS

**Fig. 3:**
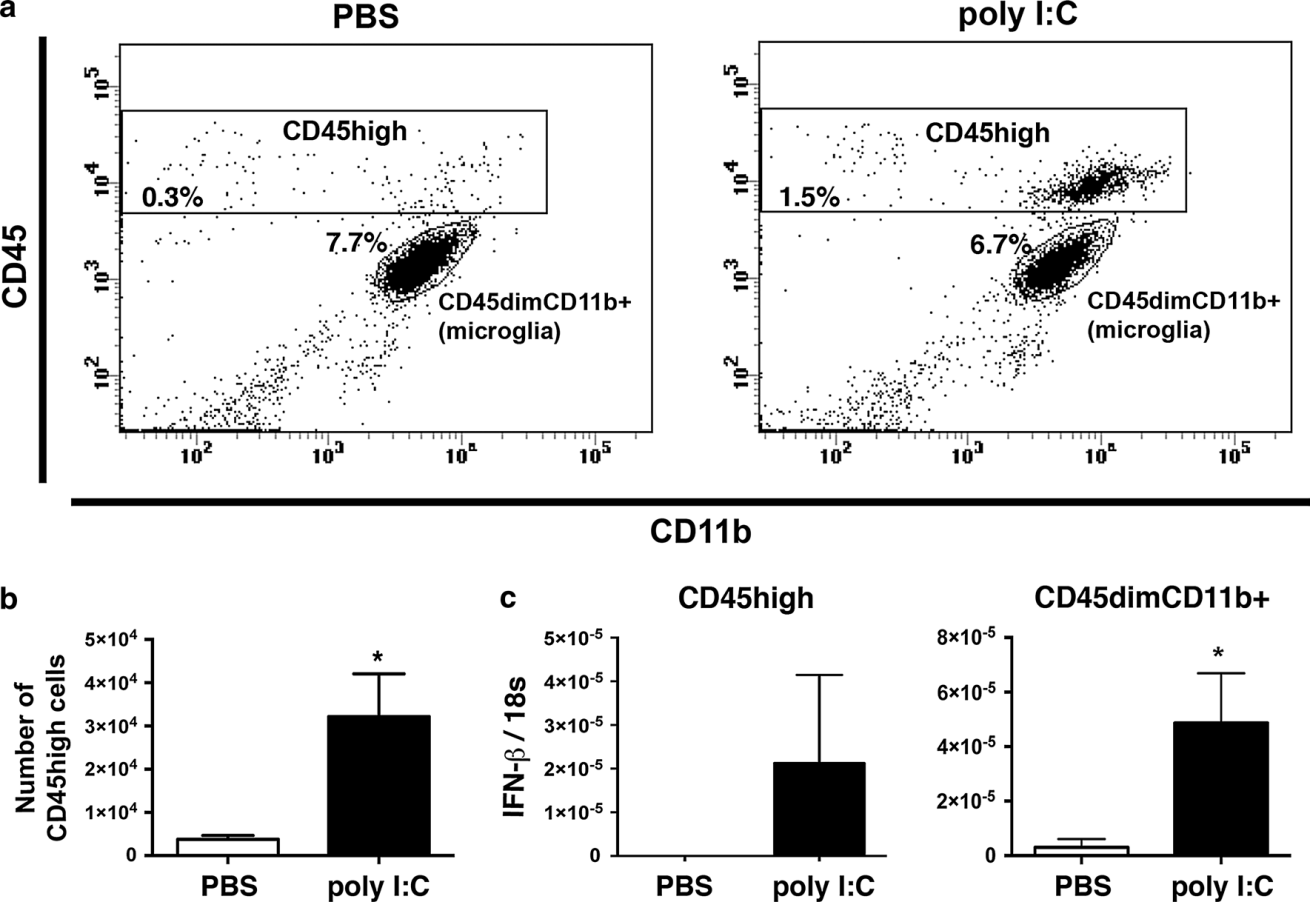
Poly I:C induced IFN-β in leukocytes. **a** A representative flow cytometry profile showing CD45^high^ (leukocytes) and CD45^dim^CD11b^+^ (microglia) cell populations isolated from the CNS of mice treated with PBS or poly I:C, 6 h previously. **b** Number of CD45^high^ cells isolated from mice treated with poly I:C (*n* = 5) compared to PBS (*n* = 3). **c** CD45^high^ (leukocytes) and CD45^dim^CD11b^+^ cells (microglia) were sorted, pooled and IFN-β mRNA was measured by qRT-PCR. *Bar graphs* show IFN-β gene expression in sorted CD45^dim^CD11b^+^ microglia (*n* = 6) from poly I:C-treated mice compared to microglia (*n* = 6) from PBS-treated mice, in which IFN-β was also detected at a low level. Intrathecal poly I:C induced detectable IFN-β expression in sorted CD45^high^ cells (*n* = 6), whereas IFN-β expression was not detected at all in sorted CD45^high^ from PBS-treated mice (*n* = 6). Data were analyzed by two-tailed nonparametric Student’s *t* test followed by Mann–Whitney test. Results are presented as mean ± SEM. *P < 0.05

Cellular source of IFN-β was further investigated using flow cytometry and cell sorting. A significant population of CD45^high^ cells was detected in perfused CNS from mice that had received intrathecal poly I:C 6 h previously (Fig. 3). Almost all of these were CD11b^+^. These are distinguished from parenchymal microglia by the lower level of CD45 staining on microglia (Fig. 3) [18]. CD45^dim^ microglia are not detected using anti-CD45 immunostaining in tissue sections by the protocol we have used. To ask whether microglia contribute to the IFN-β response and to confirm that the CD45^high^ cells included the IFN-β-producing cells identified in YFP reporter mice, CD45^dim^CD11b^+^ cells (microglia) and CD45^high^ (leukocytes) were sorted and IFN-β mRNA was measured by qRT-PCR. Intrathecal poly I:C induced increased IFN-β expression over undetectable levels in sorted CD45^high^ cells. Importantly, there was an approximate fourfold induction over already detectable levels of IFN-β mRNA expression in CD45^dim^CD11b^+^ microglia, and this was statistically significant (Fig. 3c). We also screened for IFN-α using a primer/probe set designed to recognize IFN-α(B+6+12+14). IFN-α mRNA was detected in one out of four samples of microglia from PBS-treated mice and in 2 out of 8 samples from poly I:C-treated mice (not shown). Thus, both extraparenchymal myeloid cells (leukocytes) and parenchymal microglia expressed IFN-α/β in response to poly I:C.

### Therapeutic effect of intrathecal poly I:C on EAE

We then asked whether induction of endogenous IFN-α/β in the CNS would affect EAE. We immunized C57BL/6 mice with MOG p35-55. Day of onset was defined as first presentation of symptoms, which in all cases was the loss of tail tonus. Mice were randomized on the day of onset with regard to treatment, and administered poly I:C or PBS into the cisterna magna. Mice were then evaluated for whether disease worsened, using conventional EAE clinical scoring.

**Fig. 4:**
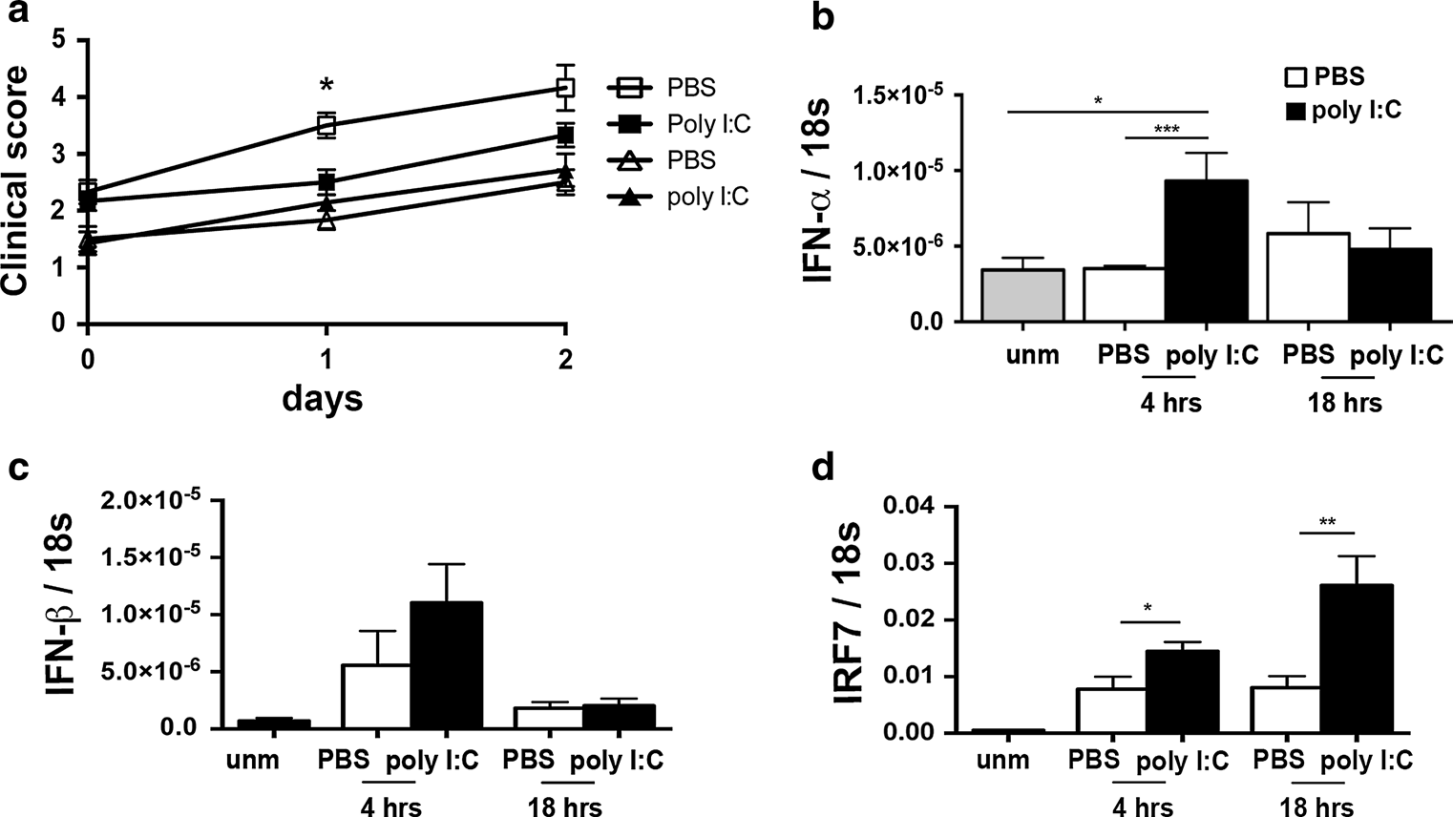
Intrathecal induction of IFN-α/β inhibited EAE. C57BL/6 and IFNAR1-deficient mice were immunized with MOG p35-55 and administered poly I:C or PBS by injection into the cisterna magna on the first day (day 0) with symptoms of EAE. **a** Shows pooled data from four independent experiments (*n* = 22). The mean clinical score did not change until the 48 h time point in mice that had received poly I:C (closed squares), whereas the control PBS-treated mice showed a significant increase in clinical score at both 24 (*P* < 0.01) and 48 h (*P* < 0.001) (*open squares*). The mean clinical score increased significantly at 24 (*P* < 0.05) and 48 h (*P* < 0.05) in IFNAR1-deficient mice (*n* = 7) that were given intrathecal poly I:C (*closed triangles*). Clinical scores at 24 h in PBS- and poly I:C-treated C57BL/6 mice were significantly different from each other (*P* < 0.05), whereas there was no difference between PBS- and poly I:C-treated IFNAR1-deficient mice at 24 h. **b** IFN-α message was significantly increased at 4 h after poly I:C treatment of mice with EAE. Mice with EAE had elevated levels of IFN-β (*P* < 0.05) (**c**) and IRF7 (*P* < 0.005) (**d**) in the CNS compared to unmanipulated (unm) mice. Levels of mRNA for IFN-β were increased at 4 h after poly I:C treatment of mice with EAE but not to significance (**c**). Levels of IRF7 message were significantly increased at 4 and 18 h post-poly I:C treatment (**d**). Data were analyzed by two-tailed nonparametric Student’s *t* test followed by Mann–Whitney test. Results are presented as mean ± SEM. *P < 0.05, **P < 0.01

The mean clinical score showed a significant increase from 24 to 48 h in PBS-treated mice, but did not change in mice that received poly I:C, until 48 h after disease onset (Fig. 4a; Supplementary Fig. 3). This transient therapeutic effect of poly I:C coincided with detection of IFN-β in reporter mice (Fig. 1a) and IFN-α/β by RT-PCR (Fig. 4a, b). Furthermore, therapeutic effect was not seen in poly I:C-treated mice lacking the IFNAR1 receptor for Type I IFN, where symptoms of disease worsened similarly to PBS-treated C57BL/6 mice (Fig. 4a).

Levels of IFN-β and IRF-7 mRNA were elevated in mice with EAE compared to healthy controls (Fig. 4c, d). Interestingly, there was no difference in IFN-α levels. Poly I:C treatment of those mice additionally increased IFN-α/β levels, although not to statistical significance for IFN-β (Fig. 4b, c). In contrast to IFN-α/β, IRF7 induction was prolonged and still increasing at 18 h after poly I:C treatment, as expected since IRF7 is an IFN target gene (Fig. 4d). A preliminary experiment with IFN-β-deficient mice showed no Type I IFN induction nor suppression of EAE by poly I:C treatment (data not shown). Thus, the alleviation of EAE due to intrathecal poly I:C treatment was accompanied by increased expression of IFN-α/β and IRF7, and dependent on IFN-α/β signaling.

### No effect of CNS-endogenous IFN-α/β on established EAE pathology

**Fig. 5:**
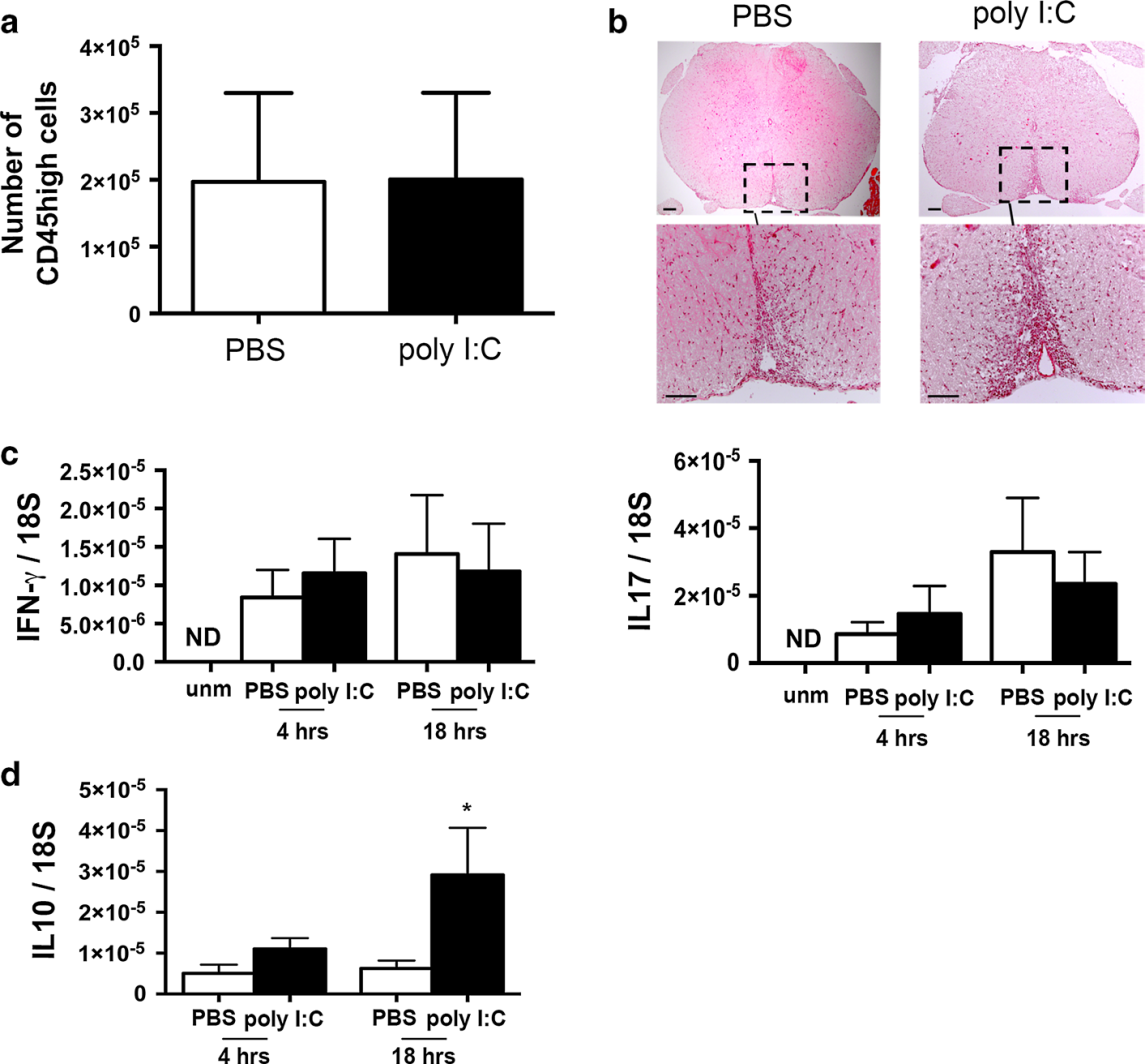
Leukocytes in CNS of mice with EAE after treatment with PBS or poly I:C. **a** Flow cytometry analysis of leukocytes (CD45^high^) in mice with EAE. Infiltration was not affected by poly I:C (*n* = 5) at 6 h. **b** Images show hematoxylin and eosin staining of spinal cord sections from mice with EAE 1 day after PBS or poly I:C treatment (original magnification is 4×). *Black boxes* show higher magnification (20×) of selected areas with infiltrates. *Scale bars* 50 µm. **c** Levels of IL-17 and IFN-γ mRNA were elevated in CNS of mice with EAE, but were not affected by poly I:C treatment at indicated times. *ND* not detected. **d** Levels of IL-10 message in CNS were significantly increased at 18 h in response to poly I:C. Number of mice was between 4 and 5 in each group

We have shown previously that lack of IRF7 exacerbates EAE and results in increased leukocyte infiltration [32]. We asked whether the protective function of endogenous IFN-α/β in EAE reflected effects on leukocyte infiltration. Mice entered the experiment with EAE and therefore showed increased numbers of CD45^high^ leukocytes in the CNS. Poly I:C treatment did not noticeably affect this infiltration 6 h after administration (Fig. 5a). Similarly, histologically detected infiltration in spinal cord showed no obvious difference between PBS and poly I:C treatment at one day (Fig. 5b).

Levels of IL-17 and IFN-γ message were elevated in the CNS of mice with EAE, and were not affected by poly I:C treatment (Fig. 5c). In contrast, IL-10 expression in CNS was significantly elevated 18 h after poly I:C injection (Fig. 5d).

### Intrathecally induced IFN-α/β stimulated glial chemokine response

**Fig. 6:**
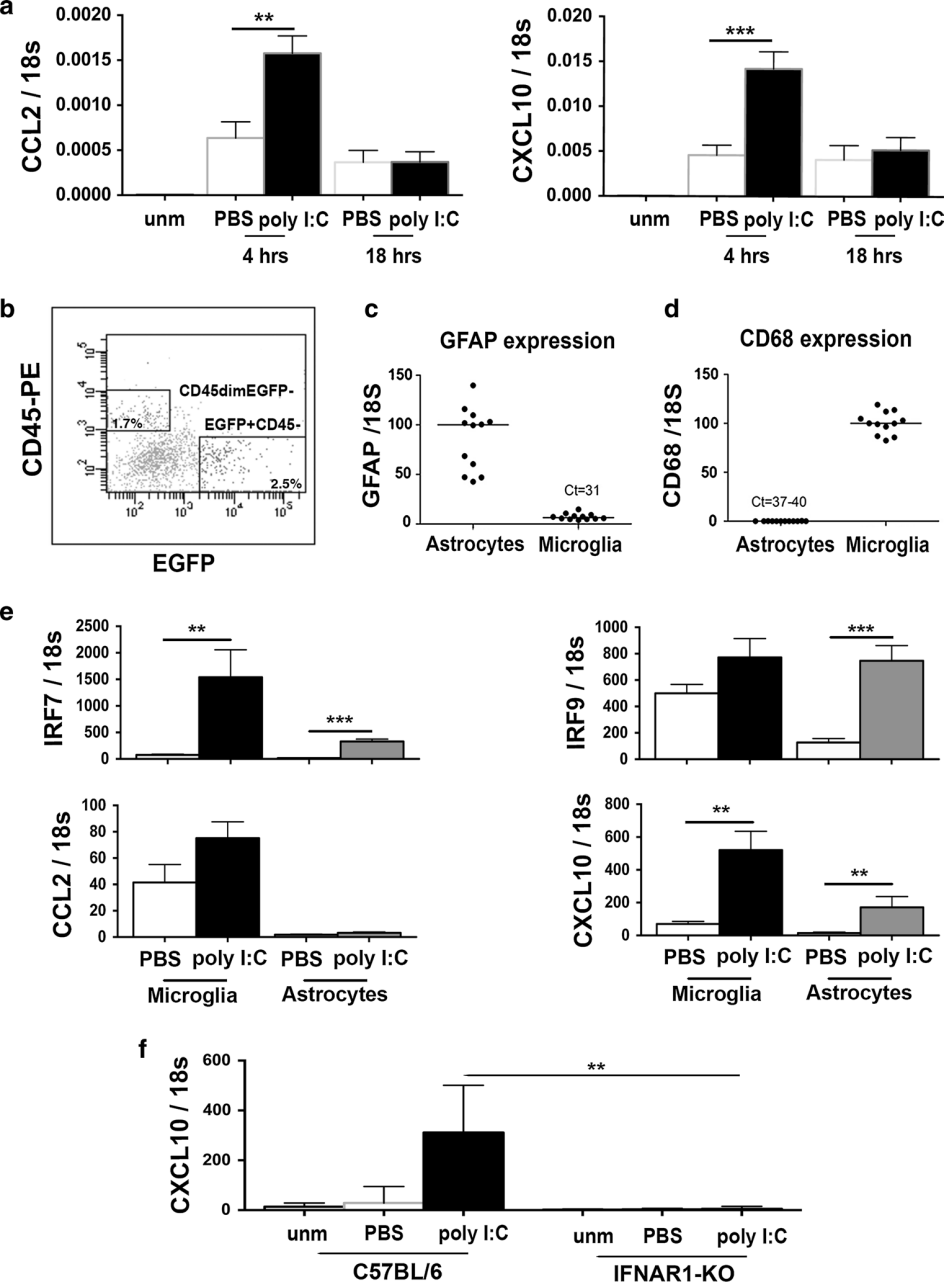
Intrathecal induction of IFN-β induced response in microglia and astrocytes. **a** Mice with EAE had elevated levels of CCL2 and CXCL10 compared to unmanipulated (unm) mice. Intrathecal administration of poly I:C transiently induced both CCL2 and CXCL10 gene expression in CNS. Expression was highest at 4 h, and reduced to levels corresponding to levels in control mice, at 18 h. **b** Representative flow cytometry profiles for cells isolated from EGFP-GFAP mice. Microglia and astrocytes were sorted as CD45^dim^EGFP^−^ and CD45^−^EGFP^+^ cells, respectively. Sorted astrocytes and microglia were validated by analysis of expression of marker genes GFAP (**c**) and CD68 (**d**), respectively. Number of mice for flow cytometry was between 6 and 12 in each group, and the experiment was repeated three times independently. **e** Intrathecal injection of poly I:C induced upregulation of IRF7 in both microglia and astrocytes. Sorted microglia from naïve mice expressed endogenously higher levels of IRF9 mRNA than astrocytes. Poly I:C induced IRF9 in astrocytes, not in microglia. Sorted microglia from naïve mice expressed endogenously higher levels of CCL2 mRNA than astrocytes. In microglia and astrocytes poly I:C did not induce changes in CCL2 mRNA expression, but induced increased CXCL10 in both microglia and astrocytes. **f** The upregulation of CXCL10 mRNA in response to poly I:C was IFNAR1 dependent. Data were analyzed by two-tailed nonparametric Student’s *t* test followed by Mann–Whitney test. Results are presented as mean ± SEM, *P < 0.05, **P < 0.01, ***P < 0.001

The chemokines CCL2 and CXCL10 are both implicated in EAE [22, 24]. They are produced by activated microglia and astrocytes, and their induction has been reported to be dependent on or associated with Type I IFN signaling [18, 36]. Expression of both chemokines was significantly increased in diseased animals (Fig. 6a). Poly I:C treatment of those mice resulted in an additional transient elevation of these two chemokines (Fig. 6a). To investigate whether this IFN-α/β-induced chemokine expression involved glial cells, we administered poly I:C intrathecally to GFAP promoter-driven EGFP reporter mice, to allow isolation of astrocytes by cell sorting (Fig. 6b, c, d). Microglia were sorted by relative CD45 levels, as before. IRF7 and IRF9 levels were elevated in astrocytes and microglia (IRF7 only) at 18 h after poly I:C injection (Fig. 6e), indicating that both glial cell types had responded to IFN-α/β. The relative IRF7 expression in response to poly I:C was higher in microglia compared to astrocytes (Fig. 6e). The IFN-α/β response was accompanied by a significant increase of CXCL10 in both astrocytes and microglia (Fig. 6e). This CXCL10 induction was not seen in IFNAR1-deficient mice (Fig. 6f), consistent with our previous study [18]. Thus, CNS-restricted IFN-α/β, from infiltrating myeloid and resident microglia, induced microglial and astroglial response and inhibited EAE.

## Discussion

We have shown that Type I IFN can be induced within the CNS and is effective against EAE, an MS-like autoimmune inflammatory disease. The cell source of IFN-α/β included parenchymal microglia and extraparenchymal myeloid cells. Expression of IL-10 was increased and IFN-α/β-dependent microglial and astrocyte response included production of the chemokine CXCL10. These findings identify the potential for CNS-endogenous immunoregulation in treatment of MS.

Many cells have the capacity to produce Type I IFN and so production of IFN-α/β in the CNS is not a priori unexpected. However, the therapeutic potential of CNS-restricted Type I IFN induction is unclear. Although Type I IFN administered in the periphery can to some level access the CNS, the effects of IFN-β therapy in MS can largely be explained as reflecting peripheral action [30]. At the same time, intra-CNS synthesis has been difficult to exclude in studies examining access of this cytokine to the CNS (see [30]). In our study, the IFN response was short-lived, reflecting a combination of stimulus-dependent activation and clearance of the ligand.

Studies of bone marrow chimeras involving lineage-specific disruption of the gene for the Type I IFN receptor, as well as adoptive transfers of encephalitogenic T cells in mice lacking IFNAR1 or IFN-β, have shown a role for IFN response within the CNS in controlling EAE [31, 35]. Prinz and colleagues [31] showed that IFN-β was selectively produced within the CNS in mice with EAE, and that response of myeloid cells (which would include microglia) was critical to protection. However, the cellular source of IFN-β within the CNS was not addressed.

Making use of sensitive reporter systems, we show that an innate immune ligand can stimulate myeloid cells in the CNS to produce IFN-β. The IFN-β/YFP+ extraparenchymal cells that we identified expressed CD11b and CD45 and therefore were likely to be CD11b^+^ DC or macrophages. The demonstration that an intrathecal stimulus can mobilize an extraparenchymal source of immunoregulatory cytokine with such rapid kinetics is striking and likely physiologically relevant.

The constitutive expression of IFN-α/β mRNA by microglia from unstimulated CNS adds to the evidence that CNS-resident microglia are active under normal circumstances [13] and that they may play a regulatory role. In a study by Prinz et al. [31], it was noticeable that IFN-β could be detected by ELISA in CNS of mice without EAE, and it was suggested that microglia could be a source. We have previously noted elevated levels of IRF7 in microglial cells [18], which can indicate both IFN production and response.

Kocur et al. (Acta Neuropathol Commun, in press) have identified microglia as major producers of endogenous IFN-β in the CNS of mice with EAE, and that IFN-β producing microglia (treated with poly I:C) mediated clearance of myelin debris in organotypic slice cultures. They also found that in vitro treatment of microglia with IFN-β regulates their myelin phagocytosis. Together their findings emphasize a protective role for IFN-β in EAE, which is in line with our findings. Unlike Kocur et al., we did not examine IFN-β/YFP mice with EAE. Intracisterna magna injection of poly I:C to otherwise unmanipulated mice induced detectable YFP in extraparenchymal myeloid cells but not in parenchymal microglia. The fourfold induction of IFN-β mRNA by poly I:C that we show in microglia was not sufficient to induce detectable YFP in reporter mice. This contrasts with Kocur et al.’s detection of peri-ventricular microglia following intracerebroventricular injection of poly I:C, which may be explained in our case by the lack of tissue trauma and accompanying glial response when using the ependymal route of injection [2, 23].

The extent to which microglial IFN-α/β contributed to IFNAR1-dependent alleviation of EAE remains to be determined. Clearly, the microglial IFN-β detected by Kocur et al. was not sufficient to prevent EAE and we must assume that the additional contribution from poly I:C-induced microglia plus extraparenchymal myeloid cells tipped the balance in our study, and IFN-α may also have had effect. Direct test of the role of IFN-α is hindered by lack of reporter or knockout animals for this multigenic family, as well as that induction of IFN-α is impaired in IFN-β-deficient mice [8, 10]. Ability to access CNS-innate IFN-driven regulatory programs represents an attractive target for potential therapy in MS and other neurological diseases. Some diseases such as Neuromyelitis optica may be worsened by IFN-β [30], but the EAE model we have studied neither involves aquaporin IV antibodies nor is dependent on an antibody response [34].

Peripheral administration of TLR3/RIG-I/RLH ligands has been shown by others to suppress or prevent EAE [7, 36, 37]. Whether poly I:C acted via TLR3 or RIG-I/MDA5 was not critical to interpretation in our study. Tzima et al. [37] demonstrated that peripheral administration of poly I:C suppressed clinical signs of EAE in mice, and that myeloid heme oxygenase-1 played a critical role in this response through IRF3 signaling and endogenous IFN-β induction. Touil et al. [36] reported that CCL2 was induced as a consequence of peripheral poly I:C injection that also suppressed EAE, and that CCL2 production was blocked after neutralization of IFN-β, arguing for Type I IFN dependence. However, that was not the case in another study, in which the chemokines CCL2 and CXCL10 were peripherally induced at increased levels compared to wild-type controls in EAE in IFNAR1-deficient mice [31]. We did not observe induction of CCL2 in microglia or astrocytes by intrathecal poly I:C, though levels were transiently increased in whole CNS. Although CCL2-deficient mice are reported to be resistant to EAE [15], transgenic overexpression of CCL2 in the CNS was reported to prevent EAE [9]. We have published that transgenic mice overexpressing CCL2 show pronounced impairment of T cell development and are consequently resistant to EAE [5]. Touil et al. [36] showed that treatment of mice with neutralizing antibody against CCL2 reversed the suppressive effect of peripherally administered poly I:C on EAE.

In a previous study of Type I IFN dependence of glial responses following synaptic degeneration in the dentate gyrus, we could show IFNAR1 dependence of CXCL10 production but not of CCL2 [18]. Whether the elevated CXCL10 that we observed here played any role in EAE amelioration was not specifically addressed. A number of reports suggest that this chemokine or signaling through its principal receptor CXCR3 may be protective in EAE, but there are also opposing data [27].

We have shown that induction of Type I IFN within the CNS has therapeutic potential against EAE, an MS-like disease. As far as we know, this is the first study to use intrathecal induction of IFN-α/β in an EAE therapeutic mode. Our findings of elevated constitutive microglial expression of IFN-α/β mRNA as well as rapid mobilization of cells producing higher levels of cytokine are consistent with a natural role for IFN-α/β in CNS homeostasis that can be exploited for therapeutic benefit. The mechanism for EAE suppression by endogenous Type I IFN is therefore of interest. It was intrinsic to our experimental design that infiltration should have initiated before induction of IFN, as would also be the case if therapeutic induction was to be considered. In contrast to peripherally applied IFN-β in MS, for which mechanism is reviewed in [30], the effect of endogenous Type I IFN is a priori unlikely to involve inhibition of leukocyte infiltration, as supported by our data showing no effect on either histologically or flow cytometrically detected infiltration. Furthermore, we did not detect any effect on the inflammatory status of encephalitogenic T cells, as measured by production of the Th1- and Th17-associated cytokines IFN-γ and IL-17, presumably because such cytokine profiles were already committed at the time of IFN induction. We consider the most likely mechanism of suppression to be via action on glial cells to modify the local milieu. Both microglia and astrocytes upregulated IRF7 early, and we showed upregulation of IL-10 as well as glial-derived CXCL10.

Interleukin-10 is implicated in suppression of MS and EAE as well as other inflammatory autoimmune pathologies, acting via regulation of cytokine, antigen presenting cell and glial response and promoting anti-inflammatory pathways [20]. Although the increase in IL-10 only became statistically significant at 18 h, it cannot be excluded as a potential regulator at earlier times. IL-10-deficient mice develop more severe EAE [4]. Guo et al. [12] have shown that IFNAR1-deficient mice had reduced IL-10 expression, and in a recent report they suggested that IFN-β may induce T cells to produce IL-10, which in turn negatively regulates Th17-associated autoimmune inflammation [39]. Type I IFN can also exert anti-inflammatory effect via the induction of IL-10 from macrophages [6]. Flow cytometry data showed that the great majority of leukocytes were myeloid cells expressing CD11b. Together with the lack of effect on IL-17 message, our results suggest that macrophages contributed to the increased level of IL-10. It may be relevant that whereas systemically administered IL-10 was only modestly effective against EAE, intranasal or intracerebroventricular delivery was more promising and included evidence of microglial involvement in disease alleviation (reviewed in [20]). More than one report has shown that the Type I IFN-dependent chemokine CXCL10 or its principal receptor CXCR3 can modulate EAE [27], and it is notable that this chemokine was induced in both microglia and astrocytes and at early time points. Our findings therefore support a role for IFN-producing microglia and macrophages and IFN-signaled glial cells.

Our findings add to evidence for a prominent microglial role in endogenous protection, both via production of Type I IFN, which we like others (Kocur et al. Acta Neuropathol Commun, in press) have now directly shown, as well as induction of candidate response modulators. An ancillary role for astrocytes as IFN-signaled cells that can also produce response modulators is likely to be important as well, especially given their strategic location at the blood–brain barrier.

The prospect for therapeutic application of such endogenous regulation of neuroinflammation will depend on identification of agonists that can access the appropriate response regulatory glial cells and sustain continued production of IFN and other regulators, sufficient to maintain neuroprotection. This is a challenge for the future.

## Electronic supplementary material

Supplementary material 1 (TIFF 18092 kb)

Supplementary material 2 (TIFF 30039 kb)

Supplementary material 3 (TIFF 10140 kb)
