# Supplementary figures and images for: Induction of endogenous Type I interferon within the central nervous system plays a protective role in experimental autoimmune encephalomyelitis
Source: Acta Neuropathol. 2015 Apr 14;130(1):107–18. doi: 10.1007/s00401-015-1418-z (PMC4469095; doi:10.1007/s00401-015-1418-z)

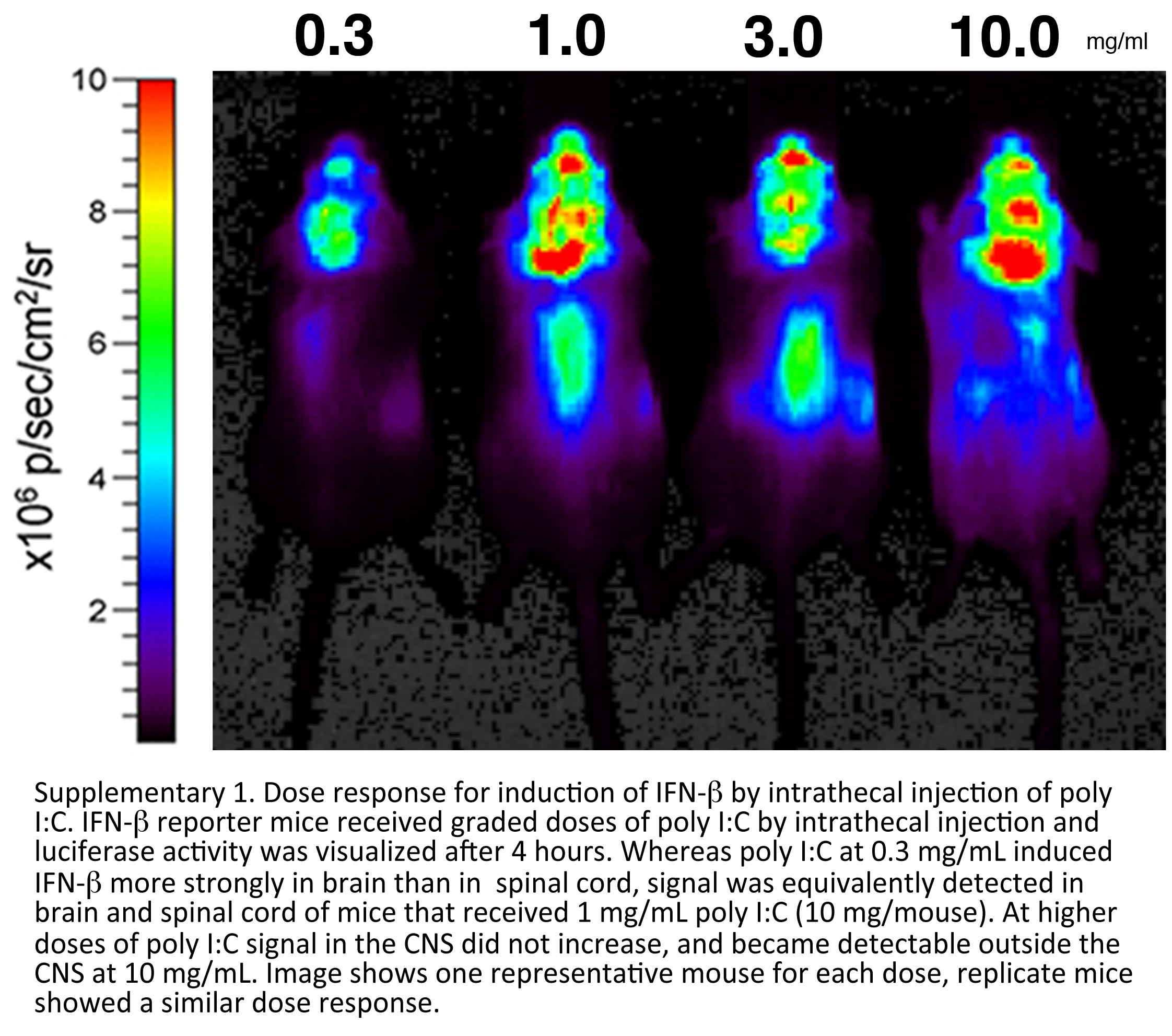

Supplement: Supplementary file 1 — Supplementary material 1 (TIFF 18092 kb) [file 401_2015_1418_MOESM1_ESM.tif]

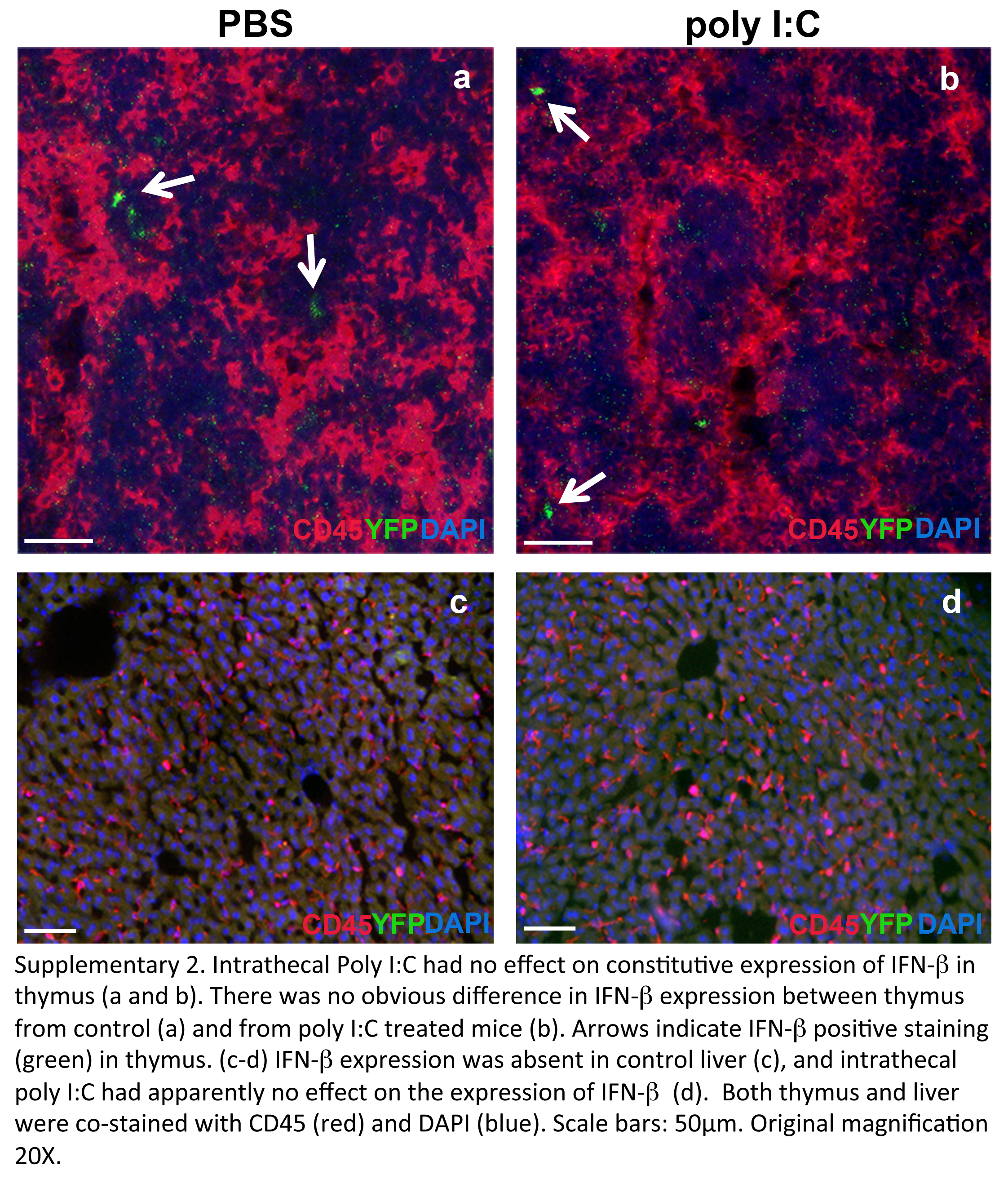

Supplement: Supplementary file 2 — Supplementary material 2 (TIFF 30039 kb) [file 401_2015_1418_MOESM2_ESM.tif]

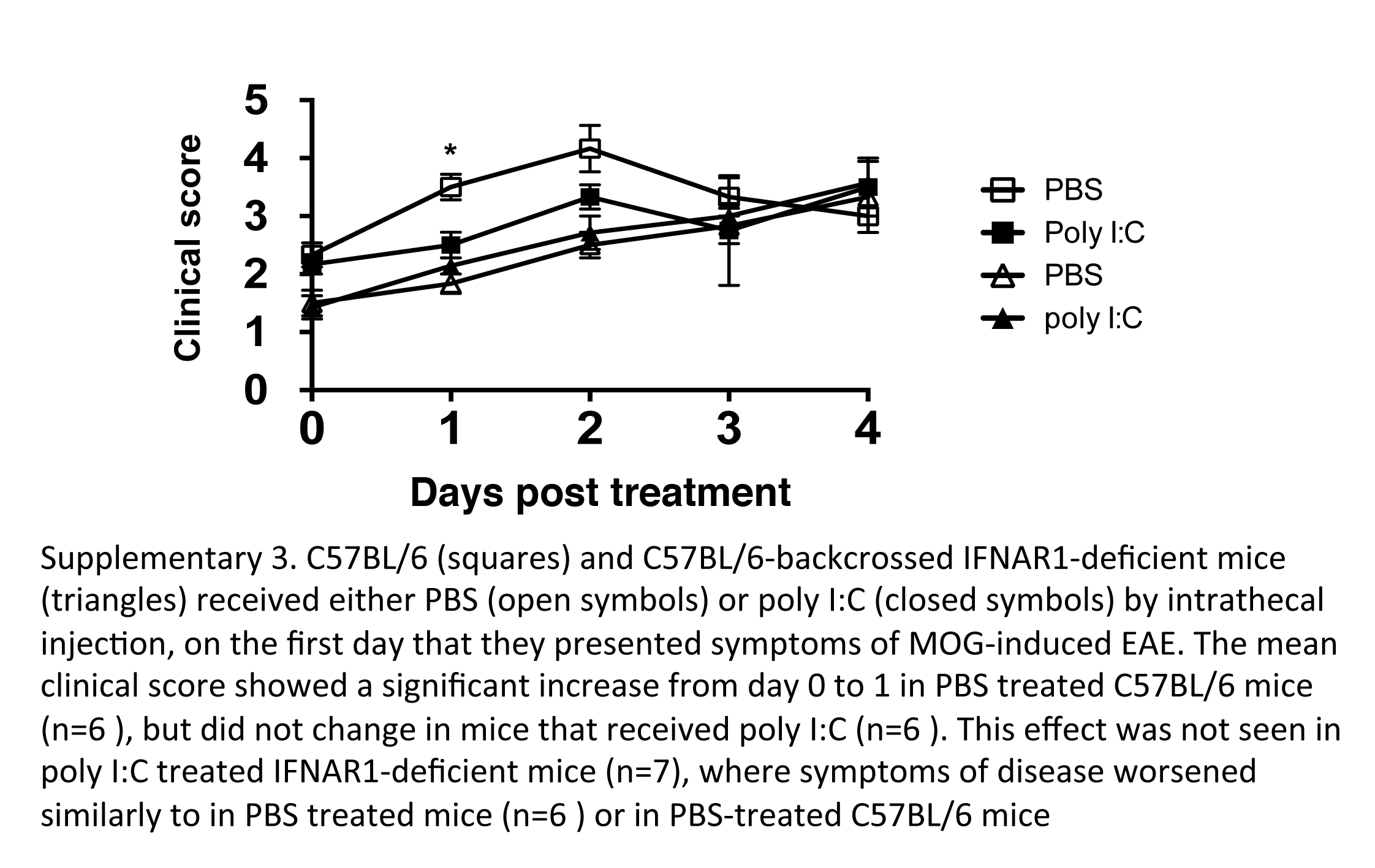

Supplement: Supplementary file 3 — Supplementary material 3 (TIFF 10140 kb) [file 401_2015_1418_MOESM3_ESM.tif]
